# Improving the performance of machine learning algorithms for health outcomes predictions in multicentric cohorts

**DOI:** 10.1038/s41598-022-26467-6

**Published:** 2023-01-19

**Authors:** Roberta Moreira Wichmann, Fernando Timoteo Fernandes, Alexandre Dias Porto Chiavegatto Filho, IACOV-BR Network, Ana Claudia Martins Ciconelle, Ana Maria Espírito Santo de Brito, Bruno Pereira Nunes, Dárcia Lima e Silva, Fernando Anschau, Henrique de Castro Rodrigues, Hermano Alexandre Lima Rocha, João Conrado Bueno dos Reis, Liane de Oliveira Cavalcante, Liszt Palmeira de Oliveira, Lorena Sofia dos Santos Andrade, Luiz Antonio Nasi, Marcelo de Maria Felix, Marcelo Jenne Mimica, Maria Elizete de Almeida Araujo, Mariana Volpe Arnoni, Rebeca Baiocchi Vianna, Renan Magalhães Montenegro Junior, Renata Vicente da Penha, Rogério Nadin Vicente, Ruchelli França de Lima, Sandro Rodrigues Batista, Silvia Ferreira Nunes, Tássia Teles Santana de Macedo, Valesca Lôbo eSant’ana Nuno

**Affiliations:** 1 School of Public Health, University of São Paulo, São Paulo, SP Brazil; 2 Brazilian Institute of Education, Development and Research-IDP, Economics Graduate Program, Brasilia, DF Brazil; 3 Fundacentro, São Paulo, SP Brazil; 4 Institute of Mathematics and Statistics, University of São Paulo, São Paulo, Brazil; 5 Instituto de Medicina, Estudos e Desenvolvimento-IMED, São Paulo, Brazil; 6 Universidade Federal de Pelotas-UFPel, Pelotas, Brazil; 7 Hospital Santa Lúcia, Divinópolis, Brazil; 8 Setor de Pesquisa da Gerência de Ensino e Pesquisa do Grupo Hospitalar Conceição, Porto Alegre, RS Brazil; 9 Programa de Pós-Graduação em Neurociências da Universidade Federal do Rio Grande do Sul, Porto Alegre, Brazil; 10 Serviço de Epidemiologia e Avaliação/Direção Geral do HUCFF/UFRJ, Rio de Janeiro, Brazil; 11 Unimed Fortaleza, Fortaleza, Ceará Brazil; 12 Departamento de Saúde Comunitária, Universidade Federal do Ceará, Fortaleza, Ceará Brazil; 13 Hospital São Francisco, Brasília, Brazil; 14 Hospital Santa Julia de Manaus, Manaus, Brazil; 15 Instituto Unimed-Rio, Universidade do Estado do Rio de Janeiro, Rio de Janeiro, Brazil; 16 Universidade de Pernambuco-UPE/UEPB, Recife, Brazil; 17 Hospital Moinhos de Vento, Porto Alegre, Brazil; 18 InRad-Institute of Radiology, School of Medicine, University of São Paulo, São Paulo, Brazil; 19 Departamento de Ciências Patológicas, Faculdade de Ciências Médicas da Santa Casa de São Paulo, São Paulo, Brazil; 20 Federal University of Amazonas, University Hospital Getulio Vargas, Manaus, AM Brazil; 21 Serviço de Controle de Infecção Hospitalar Santa Casa de São Paulo, São Paulo, Brazil; 22 Complexo Hospitalar da Universidade Federal do Ceará-EBSERH, Fortaleza, Brazil; 23 Hospital Evangélico de Vila Velha, Vila Velha, Brazil; 24 Hospital Santa Catarina de Blumenau, Blumenau, Brazil; 25 Faculdade de Medicina, Universidade Federal de Goiás, Goiânia, Goiás Brazil; 26 Secretaria de Estado da Saúde de Goiás, Goiânia, Goiás Brazil; 27 Fundação Santa Casa de Misericórdia do Pará-FSCMP, Belém, Brazil; 28 Mestrado Profissional em Gestão e Saúde na Amazônia, Belém, Brazil; 29 Escola Bahiana de Medicina e Saúde Pública, Salvador, Brazil; 30 Hospital Português da Bahia, Salvador, Brazil

**Keywords:** Health care, Prognosis, Public health, Computational models

## Abstract

Machine learning algorithms are being increasingly used in healthcare settings but their generalizability between different regions is still unknown. This study aims to identify the strategy that maximizes the predictive performance of identifying the risk of death by COVID-19 in different regions of a large and unequal country. This is a multicenter cohort study with data collected from patients with a positive RT-PCR test for COVID-19 from March to August 2020 (n = 8477) in 18 hospitals, covering all five Brazilian regions. Of all patients with a positive RT-PCR test during the period, 2356 (28%) died. Eight different strategies were used for training and evaluating the performance of three popular machine learning algorithms (extreme gradient boosting, lightGBM, and catboost). The strategies ranged from only using training data from a single hospital, up to aggregating patients by their geographic regions. The predictive performance of the algorithms was evaluated by the area under the ROC curve (AUROC) on the test set of each hospital. We found that the best overall predictive performances were obtained when using training data from the same hospital, which was the winning strategy for 11 (61%) of the 18 participating hospitals. In this study, the use of more patient data from other regions slightly decreased predictive performance. However, models trained in other hospitals still had acceptable performances and could be a solution while data for a specific hospital is being collected.

## Introduction

Around 457 million cases and 6 million deaths have been caused by COVID-19 worldwide by March 2022^1^. Nearly 29 million cases and 654 thousand deaths occurred only in Brazil, ranking third in confirmed cases and deaths. Several machine learning algorithms have been proposed for predicting COVID-19 diagnosis^2–4^ and prognosis^5–8^, with different input data such as image or laboratorial exams^9^.

In countries with large socioeconomic inequalities and different access to healthcare and resource heterogeneity^10,11^, the best strategy for selecting training data for machine learning algorithms is still unknown. While more data may improve the ability of machine learning algorithms to identify detailed pathways linking the predictors to the outcome of interest, it may also introduce noise, as new learned pathways may not be locally replicable.

Also, collecting a large number of variables may be cost prohibitive for some hospitals, and different data collection protocols between hospitals can make this aggregation unfeasible. As the use of machine learning algorithms rapidly advances in healthcare, it will be increasingly important to identify how to improve the generalization of these algorithms in different regions.

In order to identify the best strategy for selecting training data to predict COVID-19 mortality, we gathered data from 18 distinct and independent hospitals (with no direct connections, such as having the same administration or using the same EMR system) from the five regions of Brazil, and tested eight different strategies for developing predictive models, starting with only local hospital data and then seven different approaches of aggregating external training data.

## Results

### Summary population characteristics

Table 1 presents the descriptive statistics regarding the individual characteristics of the patients. The sample of the study (8477 patients with COVID-19) was mostly comprised by men (55.1%). The most common race was white (62%), although the majority (64.6%) did not provide a self-declared race. Average age was 58.4 years and patients stayed 14 days on average. Patients that died during hospital stay were older (mean age 66.7 vs. 55.2 for survivors) and were more likely to be males (60.0% vs. 53.3% for survivors). List of participants and descritptive statistics for each hospital can be found on Supplementary Tables S1 and S2 respectively.

**Table 1:** Descriptive statistics of the demographics characteristics of the sample.

Variable	Death		Total
	No	Yes	
	Mean (SD)	Mean (SD)	Mean (SD)
**Age (years)**	55.2(17.0)	66.7(15.1)	58.4(17.3)
SouthEast	57.0(15.8)	66.0(14.3)	60.3(15.9)
NorthEast	52.6(18.3)	71.2(15.3)	54.6(19.0)
Midwest	56.9(15.9)	64.3(16.7)	60.4(16.7)
South	55.2(17.1)	76.2(14.0)	58.0(18.2)
North	54.0(16.1)	68.6(14.4)	56.7(16.8)
**Hospital time**	13.2(17.3)	16.4(16.5)	14.2(17.1)
SouthEast	16.3(19.4)	16.5(14.8)	16.4(17.8)
NorthEast	6.7(11.2)	14.3(10.9)	8.0(12.0)
Midwest	13.2(13.5)	13.9(18.2)	13.5 (15.9)
South	10.0(13.6)	31.7(35.1)	12.1(18.0)
North	11.1(13.7)	20.2(23.1)	12.8(16.3)
**Male**	53.3	60.0	55.1
SouthEast	55.7	61.6	57.8
NorthEast	48.3	50.8	48.6
Midwest	55.6	60.1	57.7
South	57.3	57.7	57.3
North	55.4	63.0	56.8
**Race—White (%)**	68.3	50.6	62.1
SouthEast	63.8	58.9	62.2
NorthEast	65.9	29.0	29.2
Midwest	10.4	30.3	27.5
South	97.4	98.2	97.5
North	13.8	0.1	0.1
**Race—Black/Mixed/Asian (%)**	31.7	49.5	38.0
SouthEast	36.2	41.1	37.8
NorthEast	70.8	70.9	70.8
Midwest	89.6	69.7	72.5
South	2.6	1.8	2.5
North	86.2	93.8	88.9

### Algorithmic performance

**Figure 1:**
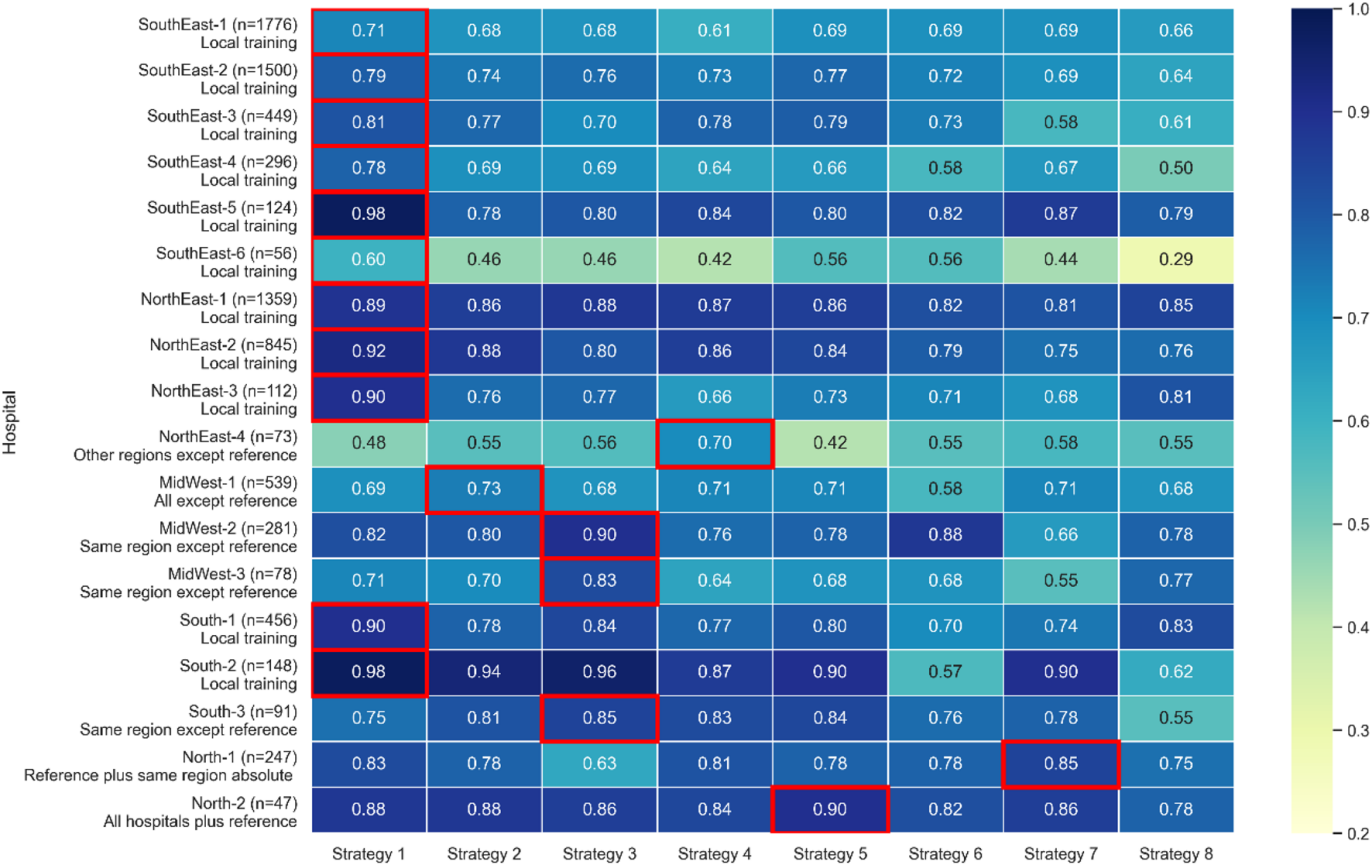
Best AUROCs according to strategy, region and hospital with the best strategy highlighted.

Figure 1 shows the results of the AUROCs for the best of the three algorithms for each strategy. Overall, the best predictive performances were obtained when using training data from the same hospital, which was the winning strategy for 11 (61%) of the 18 participating hospitals.

**Figure 2:**
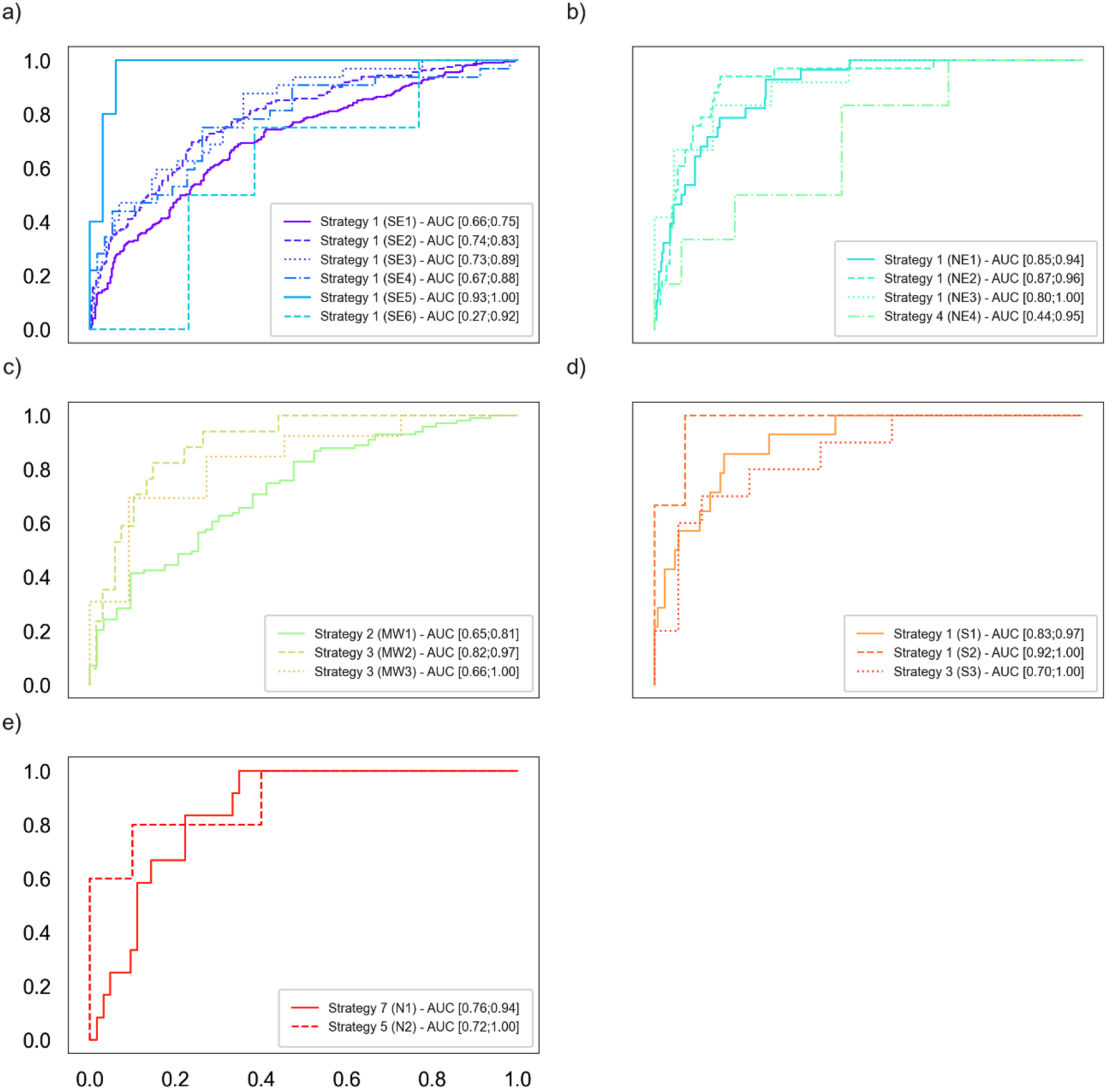
AUROCs of the winning strategy per region. (**a**) Southeast, (**b**) Northeast, (**c**) Midwest, (**d**) South, (**e**) North.

Figure 2 presents the AUROCs of the winning strategy for each hospital, separated by regions. For the southeast region, the most populous region of Brazil and where most of the data was collected, the winning strategy for every hospital was training with only local data. Supplementary Figs. S2 and S3 show recall and specificities from best strategies.

Table 2 presents a summary of the best algorithm for each strategy. Overall, extreme gradient boosting (XGBoost) was the algorithm that presented the highest number of winning predictive performances regarding AUROCs (67/144, 46.5%), followed closely by Light GBM with 61 (42.4%) and catboost with 16 (11.1%). The list of the final hyperparameters for each algorithm is available in Supplementary Table S3. Calibration for best models are presented in Supplementary Table S4.

**Table 2:** Algorithm with the best predictive performance per strategy.

	Strategy								Total
	1	2	3	4	5	6	7	8	
Catboost	6 (33.3%)	1 (5.6%)	1 (5.6%)	0 (0.0%)	1 (5.6%)	2 (11.1%)	2 (11.1%)	3 (16.7%)	16 (11.1%)
LightGBM	5 (27.8%)	12 (66.7%)	11 (61.1%)	10 (55.6%)	8 (44.4%)	4 (22.2%)	7 (38.9%)	4 (22.2%)	61 (42.4%)
XGBoost	7 (38.9%)	5 (27.8%)	6 (33.3%)	8 (44.4%)	9 (50%)	12 (66.7%)	9 (50.0%)	11 (61.1%)	67 (46.5%)
Total	18	18	18	18	18	18	18	18	144

## Discussion

We found that the different strategies for training data selection were able to predict COVID-19 mortality with good overall performance, using only routinely-collected data, with an AUROC of 0.7 or higher per strategy, with few exceptions. The best overall strategy was training and testing using only the reference hospital data, achieving the highest predictive performance in 11 of the 18 different hospitals.

In this study, while in some cases adding more data from different hospitals and regions improved predictive performance, in most scenarios it decreased the predictive ability of the algorithms. The inclusion of data from other hospitals contributed to training data noise possibly due to heterogeneity in hospital practices^12^ and in most cases deteriorated the predictive performance as seen in other studies^13,14^, possibly due to different patient demographics, and variable interactions that are not locally reproductible^15^. Other studies that included data from different hospitals and found high predictive performance may have benefited from using data from connected hospitals with similar patients using different techniques or larger samples^16–18^. Our study is unique in the sense that we analyzed data from 18 independent hospitals from all the five regions of a large and unequal country.

This study has some limitations that need to be acknowledged. First, even though we analyzed hospitals from every region of Brazil, they were not equally distributed, with a higher number of patients from the southeast and northeast regions, which are also the most populous. Another limitation is that as the 18 hospitals were unconnected and independent, there may have been differences on local data collection procedures and sample size that influenced the final results. Finally, some hospitals had small samples, but were included for aggregating purposes with other regions to check if other strategies improved overall performance.

In conclusion, we found that using only hospital data can yield better predictive results when compared to adding data from other regions with different population and socioeconomic characteristics. We found that algorithms trained with data from other hospitals frequently decreased local performance even if it considerably increased the training data available. However, models trained with data from other hospitals still presented acceptable performances, and could be an option while data for a specific hospital is still being collected.

## Methods

### Data source

A cohort of 16,236 patients from 18 distinct hospitals of all regions of Brazil were followed between March and August 2020. The map with the geographic location of participating hospitals is available in Supplementary Fig. S1. We filtered only adult patients (> 18 years) with a positive RT-PCR diagnostic exam for COVID-19, resulting in 8477 patients. Of these, 2356 (28%) died as a result of complications caused by COVID-19. The mortality outcome referred to the current hospital admission for COVID-19, independently of the timeframe. Hospitalization was only analyzed at the time of COVID-19 diagnosis and further hospitalizations of the patient were not included in the study. We used as predictors only variables collected in early hospital admission, i.e. within 24 h before and 24 h after the RT-PCR exam. The full list of hospitals is available in Supplementary Table S1.

**Figure 3:**
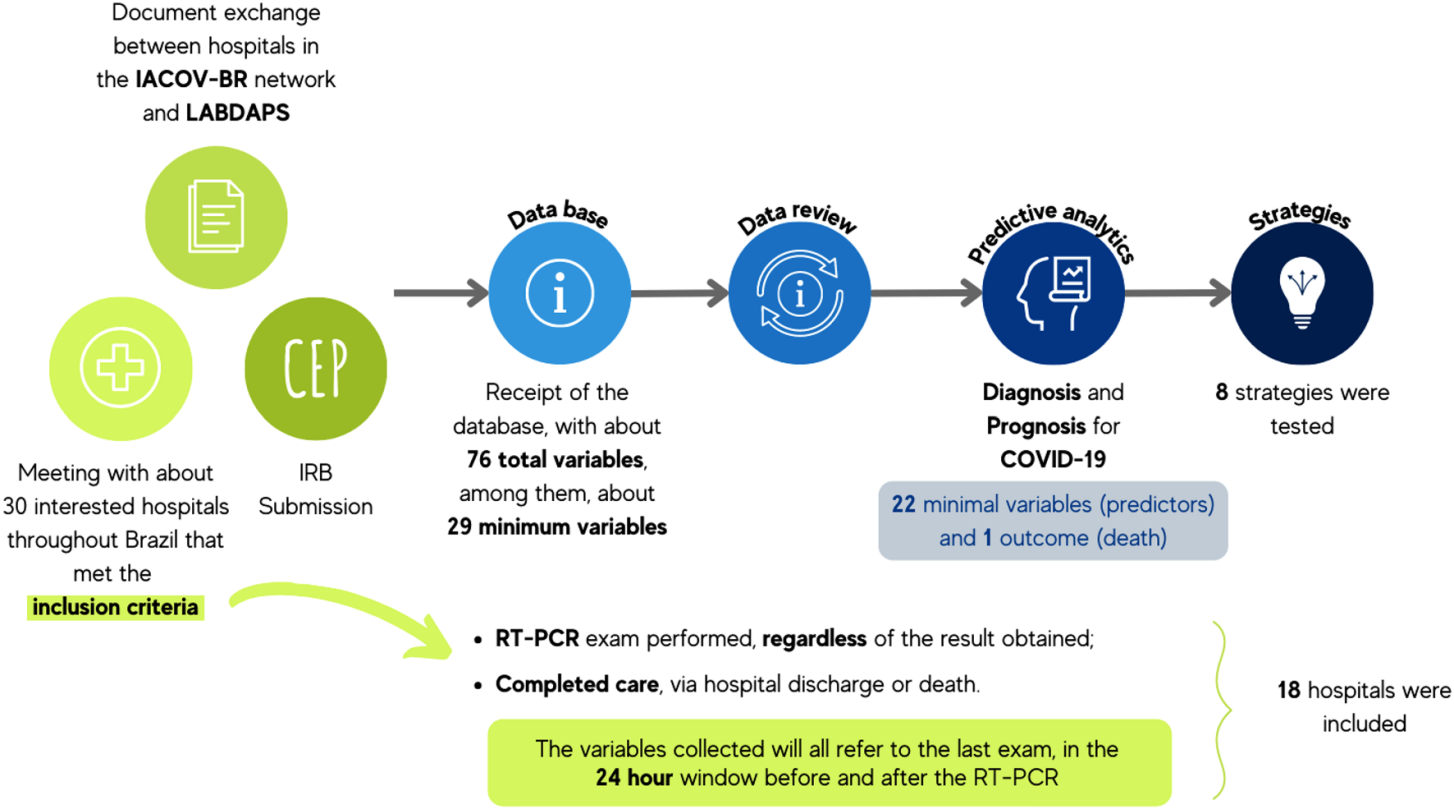
Process overview. From inclusion criteria to feature selection.

A total of 22 predictors were selected among routinely-collected variables in all hospitals, including age, sex, heart rate, respiratory rate, systolic pressure, diastolic pressure, mean pressure, temperature, hemoglobin, platelets, hematocrit, red cells count, mean corpuscular hemoglobin (mch), red cell distribution width (rdw), mean corpuscular volume (mcv), leukocytes, neutrophil, lymphocytes, basophils, eosinophils, monocytes and C-reactive protein. Figure 3 illustrates the overall process.

The study was approved by the Institutional Review Board (IRB) of the University of São Paulo (CAAE: 32872920.4.1001.5421), which included a waiver of informed consent. The data and the partnership with all members of IACOV-BR are included in this approval. The study followed the guidelines of the Transparent Reporting of a Multivariable Prediction Model for Individual Prognosis or Diagnosis (TRIPOD)^19^.

### Machine learning techniques

Three popular machine learning models for structured data (lightGBM^20^ catboost^21^, and extreme gradient boosting^22^) were trained to predict COVID-19 mortality using routinely-collected data. Eight different strategies were tested to identify the best data selection strategy for each hospital and each of the three algorithms.

### Strategies and preprocessing techniques

Initially, we used a single hospital data as the baseline strategy, splitting the data in 70% for training and 30% for testing, with the latter used to predict mortality risk. We then also tested seven different data aggregation strategies to assess the performance of the algorithms with different training data, as presented in Table 3.

**Table 3:** Clustering strategies for training and testing.

Strategy	Description
1. Local training	Training with 70% of a single hospital data and testing on the other 30%
2. All hospitals except reference	Training using 70% of all hospitals data excluding the reference hospital, and testing on 30% of the reference hospital data
3. Same region except reference	Training using 70% of all hospitals data in the same geographic region excluding the reference hospital, and testing on 30% of the reference hospital data
4. Other regions except reference	Training using 70% of all hospitals data in other geographic regions and testing on 30% of the reference hospital data
5. All hospitals plus reference	Training using 70% of all hospitals data and 70% of the reference hospital, and testing on 30% of the reference hospital data
6. Reference plus the all hospitals absolute number	Training using 70% of the reference hospital data plus the same absolute number of patients of all hospitals, and testing on 30% of the reference hospital data
7. Reference plus the same region absolute number	Training using 70% of the reference hospital data plus the same absolute number of patients of hospitals from the same region, and testing on 30% of the reference hospital data
8. Reference plus the other regions absolute number	Training using 70% of the reference hospital data plus the same absolute number of patients of hospitals from other regions, and testing on 30% of the reference hospital data

Variables with more than two categories were represented by a set of dummy variables, with one variable for each category. Continuous variables were standardized using the z-score. Variables with a correlation greater than 0.90 were discarded. Variables with more than 90% missing data were also discarded. Remaining variables with missing data were first imputed by the median. We also analyzed the use of the multiple imputation by chained equation (MICE)^23^ technique, but it did not improve the predictive performance of the models (Supplementary Fig. S4). We used K-fold cross-validation with 10 folds with Bayesian optimization (HyperOpt) to select the hyperparameters. Random oversampling was performed in the training set to improve class imbalance while keeping the test set intact^24^.

To evaluate the performance of the algorithms, we calculated the following metrics for each strategy: accuracy, recall (sensitivity), specificity, positive predictive value (PPV or precision), negative predictive value (NPV) and F1 score. The area under the receiver operating characteristic curve (AUROC) was the main metric used to select the best model among the different scenarios. All the results reported in this study are from the test set. Confidence intervals for AUROC curves were estimated using Delong method for computing the covariance of unadjusted AUC.

### Institutional review board statement

The name of the ethics committee is “Comitê de Ética em Pesquisa da Faculdade de Saúde Pública da USP”. All the study protocol was approved by this Committee following all methods in accordance with the relevant guidelines and regulations. The approval date of the project was June 2020.

## Supplementary Information

Supplementary Information.

## Data Availability

The data comes from 18 distinct hospitals, and it is not publicly available as it contains information of patients in accordance with the Brazilian data protection law (Lei Geral de Proteção de Dados nº 13.709/2018) but are available from the corresponding author on reasonable request.

## Code availability

All the code written to develop the models can be found on https://github.com/labdaps/iacov_br_public.

## References

[1] Worldometers. COVID Live - Coronavirus Statistics [Internet]. [cited 2022 Mar 13]. Available from: https://www.worldometers.info/coronavirus/.

[2] Canas LS, Sudre CH, Capdevila Pujol J, Polidori L, Murray B, Molteni E (2021). Early detection of COVID-19 in the UK using self-reported symptoms: A large-scale, prospective, epidemiological surveillance study. Lancet Digit Heal..

[3] Batista, A. F. M., Miraglia, J. L., Donato, H. R., & Chiavegatto Filho, A. D. P. COVID-19 diagnosis prediction in emergency care patients: A machine learning approach. medRxiv. 2020.

[4] Soltan AAS, Yang J, Pattanshetty R, Novak A, Yang Y, Rohanian O (2022). Real-world evaluation of rapid and laboratory-free COVID-19 triage for emergency care: external validation and pilot deployment of artificial intelligence driven screening. Lancet Digit. Heal..

[5] Fernandes FT, de Oliveira TA, Teixeira CE, Batista AFM, Dalla Costa G, Chiavegatto Filho ADP (2021). A multipurpose machine learning approach to predict COVID-19 negative prognosis in São Paulo. Brazil. Sci. Rep..

[6] Chieregato, M., Frangiamore, F., Morassi, M., Baresi, C., Nici, S., & Bassetti, C. *et al.* A hybrid machine learning/deep learning COVID-19 severity predictive model from CT images and clinical data. *Sci. Rep.* 1–15 (2021). Available from: http://arxiv.org/abs/2105.06141. 10.1038/s41598-022-07890-1 PMC8919158 35288579

[7] Kamran F, Tang S, Otles E, McEvoy DS, Saleh SN, Gong J (2022). Early identification of patients admitted to hospital for covid-19 at risk of clinical deterioration: Model development and multisite external validation study. BMJ.

[8] Murri R, Lenkowicz J, Masciocchi C, Iacomini C, Fantoni M, Damiani A (2021). A machine-learning parsimonious multivariable predictive model of mortality risk in patients with Covid-19. Sci. Rep..

[9] Wynants L, Van Calster B, Collins GS, Riley RD, Heinze G, Schuit E (2020). Prediction models for diagnosis and prognosis of covid-19: Systematic review and critical appraisal. BMJ.

[10] Albuquerque MV, d’Ávila VAL, Lima LD, Ferreira MP, Fusaro ER, Iozzi FL (2017). Regional health inequalities: Changes observed in Brazil from 2000–2016. Cienc e Saude Coletiva..

[11] Souza Noronha KVM, Guedes GR, Turra CM, Andrade MV, Botega L, Nogueira D (2020). The COVID-19 pandemic in Brazil: Analysis of supply and demand of hospital and ICU beds and mechanical ventilators under different scenarios. Cad Saude Publica..

[12] Kelly CJ, Karthikesalingam A, Suleyman M, Corrado G, King D (2019). Key challenges for delivering clinical impact with artificial intelligence. BMC Med..

[13] Wong A (2021). External validation of a widely implemented proprietary sepsis prediction model in hospitalized patients. JAMA Intern. Med..

[14] Roimi M (2020). Early diagnosis of bloodstream infections in the intensive care unit using machine-learning algorithms. Intensive Care Med..

[15] Futoma J, Simons M, Panch T, Doshi-Velez F, Celi LA (2020). The myth of generalisability in clinical research and machine learning in health care. Lancet Digit. Heal..

[16] Dou Q, So TY, Jiang M, Liu Q, Vardhanabhuti V, Kaissis G (2021). Federated deep learning for detecting COVID-19 lung abnormalities in CT: A privacy-preserving multinational validation study. NPJ Digit. Med..

[17] Salam MA, Taha S, Ramadan M (2021). COVID-19 detection using federated machine learning. PLoS ONE.

[18] Dayan I, Roth HR, Zhong A, Harouni A, Gentili A, Abidin AZ (2021). Federated learning for predicting clinical outcomes in patients with COVID-19. Nat. Med..

[19] Moons KGM, Altman DG, Reitsma JB, Ioannidis JPA, Macaskill P, Steyerberg EW (2015). Transparent reporting of a multivariable prediction model for individual prognosis or diagnosis (TRIPOD): Explanation and elaboration. Ann. Intern. Med..

[20] Ke G, Meng Q, Finley T, Wang T, Chen W, Ma W (2017). Lightgbm: A highly efficient gradient boosting decision tree. Adv. Neural. Inf. Process Syst..

[21] Dorogush, A. V., Ershov, V., & Gulin, A. CatBoost: gradient boosting with categorical features support. CoRR [Internet]. 2018;abs/1810.1. Available from: http://arxiv.org/abs/1810.11363.

[22] Chen, T., & Guestrin, C. XGBoost: A Scalable Tree Boosting System. In *KDD ’16 Proceedings of the 22nd ACM SIGKDD International Conference on Knowledge Discovery and Data Mining [Internet]*. ACM (2016). 10.1145/2939672.2939785.

[23] van Buuren S, Groothuis-Oudshoorn K (2011). mice: Multivariate Imputation by chained equations in R. J. Stat. Softw..

[24] He H, Ma Y (2013). Imbalanced learning: foundations, algorithms, and applications.

