# Supplementary material for: Improving the performance of machine learning algorithms for health outcomes predictions in multicentric cohorts
Source: Sci Rep. 2023 Jan 19;13:1022. doi: 10.1038/s41598-022-26467-6 (PMC9849836; doi:10.1038/s41598-022-26467-6)
Supplement: Supplementary file 1 — Supplementary Information. [file 41598_2022_26467_MOESM1_ESM.docx]

**Improving the performance of machine learning algorithms for health outcomes predictions in multicentric cohorts**

***Roberta Moreira Wichmann, Ph.D^1,2^**

**Fernando Timoteo Fernandes, Ph.D ^1,3^**

**Alexandre Dias Porto Chiavegatto Filho, Ph.D ^1^**

**IACOV-BR Network^1^**

**^1^ University of São Paulo, School of Public Health, São Paulo, SP, Brazil**

**^2^ Brazilian Institute of Education, Development and Research – IDP. Economics Graduate Program, Brasilia, DF, Brazil.**

**^3^ Fundacentro, São Paulo, SP, Brazil**

**Correspondence to: roberta.wichmann@idp.edu.br**

**Supplementary Table 1 -** List of participating hospitals.

| **Hospital** | **CNES** | **State** | **Type of legal entity** | **Level of government** | **Type of hospital** | **Delivery** | **Teaching and research** | **N. of beds** |
| --- | --- | --- | --- | --- | --- | --- | --- | --- |
| Hospital Universitário Getúlio Vargas | 2017644 | AM | Government-owned corporation | Federal | General hospital | Tertiary care | Teaching Hospital | 246 |
| Hospital Santa Julia | 2018055 | AM | Business entity | State | General hospital | Tertiary care | Has no teaching activity | 176 |
| Real Sociedade Portuguesa de Beneficência Dezesseis De Setembro - Hospital Português da Bahia | 0004251 | BA | Not-for-profit | State and municipal | General hospital | Tertiary care | Teaching Hospital | 344 |
| Hospital Unimed Fortaleza | 3242587 | CE | Business entity | State | General hospital | Tertiary care | Teaching Hospital | 400 |
| Hospital Universitário Walter Cantídio - Complexo Hospitalar da Universidade Federal do Ceará - EBSERH | 2561492 | CE | Government-owned corporation | Federal | General hospital | Tertiary care | Teaching Hospital | 197 |
| Hospital Universitário HC | 0000396 | PE | Business entity | State | General hospital | Tertiary care | Teaching Hospital | 290 |
| Hospital Estadual de Luziânia - HEL | 2340429 | GO | Government-owned corporation | State | General hospital | Tertiary care | Has no teaching activity | 60 |
| Hospital Estadual de Trindade - HETRIN | 5095808 | GO | Government-owned corporation | State | General hospital | Tertiary care | Has no teaching activity | 60 |
| Hospital Santa Lúcia | 2815966 | DF | Business entity | State | General hospital | Tertiary care | Has no teaching activity | 418 |
| Hospital Universitário Clementino Fraga Filho | 2280167 | RJ | Government-owned corporation | Federal | General hospital | Tertiary care | Teaching Hospital | 364 |
| Hospital Unimed-Rio | 7251491 | RJ | Business entity | Municipal | General hospital | Tertiary care | Teaching Hospital | 192 |
| Hospital Santa Casa de São Paulo | 2688689 | SP | Not-for-profit | State | General hospital | Tertiary care | Teaching Hospital | 568 |
| Hospital São Francisco de Mogi Guaçú | 2081016 | SP | Business entity | Municipal | General hospital | Secondary care | Has no teaching activity | 138 |
| Hospital Evangélico de Vila Velha - HEVV | 2494442 | ES | Not-for-profit | State | General hospital | Tertiary care | Has no teaching activity | 187 |
| Hospital das Clínicas da Faculdade de Medicina da USP - HCFMUSP | 2078015 | SP | Government-owned corporation | State | General hospital | Tertiary care | Teaching Hospital | 2500 |
| Hospital Santa Catarina Blumenau | 2522101 | SC | Not-for-profit | Municipal | General hospital | Tertiary care | Has no teaching activity | 153 |
| Hospital Moinhos de Vento | 3006522 | RS | Not-for-profit | Municipal | General hospital | Tertiary care | Has no teaching activity | 445 |
| Hospital Escola da UFPel | 2252694 | RS | Government-owned corporation | Municipal | General hospital | Tertiary care | Teaching Hospital | 161 |

**Supplementary Table 2 -** Descriptive statistics of the demographics characteristics per Hospital

|  | **Death** | |  |
| --- | --- | --- | --- |
| **Variable** | **No** | **Yes** | **Total** |
|  | **Mean (SD)** | **Mean (SD)** | **Mean (SD)** |
| Age (years) - All | 55.2(17.0) | 66.7(15.1) | 58.4(17.3) |
| SE-1 | 56.2(14.9) | 64.0(14.2) | 59.5(15.1) |
| SE-2 | 55.5(15.9) | 66.2(13.6) | 59.3(16.0) |
| SE-3 | 62.7(16.9) | 77.5(11.6) | 66.2(17.0) |
| SE-4 | 60.3(14.3) | 66.6(14.1) | 62.6(14.6) |
| SE-5 | 51.7(14.8) | 63.2(16.1) | 53.3(15.5) |
| SE-6 | 66.3(17.9) | 69.1(16.1) | 67.0(17.5) |
| NE-1 | 47.6(17.1) | 74.0(14.7) | 49.4(18.2) |
| NE-2 | 61.0(17.6) | 73.3(14.3) | 62.6(17.7) |
| NE-3 | 53.6(17.0) | 62.9(16.1) | 57.0(17.3) |
| NE-4 | 52.8(15.7) | 64.3(13.9) | 56.2(16.0) |
| MW-1 | 52.8(14.0) | 61.3(16.0) | 58.0(15.8) |
| MW-2 | 61.5(16.5) | 81.5(9.7) | 65.6(17.4) |
| MW-3 | 52.0(14.2) | 64.2(15.9) | 58.6(16.3) |
| S-1 | 57.4(16.7) | 83.5(8.2) | 60.1(17.9) |
| S-2 | 47.7(16.4) | 76.6(12.3) | 49.6(17.7) |
| S-3 | 57.5(16.0) | 65.9(14.5) | 60.7(16.0) |
| N-1 | 53.7(16.1) | 72.6(12.8) | 56.6(17.0) |
| N-2 | 56.2(16.3) | 59.1(13.4) | 57.1(15.4) |
| Hospital time | 13.2(17.3) | 16.4(16.5) | 14.2(17.1) |
| SE-1 | 15.1(21.6) | 15.4(14.0) | 15.2(18.8) |
| SE-2 | 19.1(17.2) | 17.0(12.6) | 18.3(15.7) |
| SE-3 | 14.1(19.5) | 20.0(19.2) | 15.5(19.6) |
| SE-4 | 13.9(17.3) | 19.5(22.7) | 15.9(19.6) |
| SE-5 | N/A | N/A | N/A |
| SE-6 | 9.3(9.4) | 7.5(7.7) | 8.8(9.1) |
| NE-1 | N/A | N/A | N/A |
| NE-2 | 4.3(6.5) | 11.9(7.4) | 5.3(7.1) |
| NE-3 | 13.3(9.2) | 14.7(8.8) | 13.8(9.1) |
| NE-4 | 32.3(27.8) | 26.3(19.2) | 30.6(25.7) |
| MW-1 | 16.9(11.6) | 12.4(12.3) | 13.9(12.3) |
| MW-2 | 12.0(14.8) | 21.5(33.9) | 14.0(20.5) |
| MW-3 | 9.4(6.7) | 11.4(7.3) | 10.5(7.1) |
| S-1 | 12.3(14.3) | 28.9(25.5) | 14.0(16.6) |
| S-2 | 3.3(8.0) | 44.8(61.5) | 6.1(20.6) |
| S-3 | N/A | N/A | N/A |
| N-1 | 9.5(11.5) | 18.8(19.0) | 11.0(13.4) |
| N-2 | 21.5(20.6) | 23.7(30.6) | 22.2(24.5) |
| Male (%) | 53.3 | 60.0 | 55.1 |
| SE-1 | 59.7 | 62.2 | 60.8 |
| SE-2 | 52.3 | 62.0 | 55.8 |
| SE-3 | 56.9 | 61.3 | 57.9 |
| SE-4 | 49.2 | 57.0 | 52.0 |
| SE-5 | 52.3 | 58.8 | 53.2 |
| SE-6 | 60.5 | 53.8 | 58.9 |
| NE-1 | 43.1 | 52.1 | 43.7 |
| NE-2 | 56.5 | 48.2 | 55.4 |
| NE-3 | 47.9 | 48.8 | 48.2 |
| NE-4 | 59.6 | 51.9 | 60.3 |
| MW-1 | 63.5 | 61.3 | 62.2 |
| MW-2 | 48.2 | 49.1 | 48.4 |
| MW-3 | 55.6 | 65.9 | 61.0 |
| S-1 | 58.4 | 53.2 | 57.9 |
| S-2 | 51.4 | 60.0 | 52.0 |
| S-3 | 63.2 | 61.8 | 62.6 |
| N-1 | 55.5 | 57.9 | 55.9 |
| N-2 | 54.8 | 75.0 | 61.7 |
| Race – White (%) | 68.3 | 50.6 | 62.1 |
| SE-1 | N/A | N/A | N/A |
| SE-2 | 61.2 | 60.5 | 60.9 |
| SE-3 | N/A | N/A | N/A |
| SE-4 | 42.3 | 27.1 | 36.8 |
| SE-5 | 92.5 | 100.0 | 93.5 |
| SE-6 | N/A | N/A | N/A |
| NE-1 | N/A | N/A | N/A |
| NE-2 | N/A | N/A | N/A |
| NE-3 | 45.1 | 41.5 | 43.8 |
| NE-4 | 0.7 | 4.8 | 6.8 |
| MW-1 | N/A | 25.0 | 15.2 |
| MW-2 | N/A | N/A | N/A |
| MW-3 | 2.8 | 11.9 | 8.9 |
| S-1 | 92.7 | 95.7 | 93.0 |
| S-2 | 84.1 | 100.0 | 85.1 |
| S-3 | N/A | N/A | N/A |
| N-1 | N/A | N/A | N/A |
| N-2 | 12.9 | 6.2 | 10.6 |
| Race – Black/Mixed/Asian (%) | 31.7 | 49.5 | 38.0 |
| SE-1 | N/A | N/A | N/A |
| SE-2 | 38.8 | 39.5 | 39.1 |
| SE-3 | N/A | N/A | N/A |
| SE-4 | 57.7 | 72.9 | 63.2 |
| SE-5 | 7.5 | 50.5 | 62.0 |
| SE-6 | N/A | N/A | N/A |
| NE-1 | N/A | N/A | N/A |
| NE-2 | N/A | N/A | N/A |
| NE-3 | 54.9 | 58.5 | 56.2 |
| NE-4 | 92.3 | 95.2 | 93.2 |
| MW-1 | N/A | 75.0 | 84.8 |
| MW-2 | N/A | N/A | N/A |
| MW-3 | 97.2 | 88.1 | 91.1 |
| S-1 | 7.3 | 4.3 | 7.0 |
| S-2 | 15.9 | 0 | 14.9 |
| S-3 | N/A | N/A | N/A |
| N-1 | N/A | N/A | N/A |
| N-2 | 87.1 | 93.8 | 89.4 |

**Supplementary Table *3* -** Hyperparameters of the best machine learning algorithms for each final hospital.

| **Hospital**  **(Strategy)** | **Best algorithm** | **Hyperparameters** |
| --- | --- | --- |
| **SE-1**  **(Local Training)** | *XGBoost* | base_score=0.5, colsample_bylevel=1, colsample_bynode=1, colsample_bytree=0.85, gamma=0.65, importance_type='gain'  learning_rate=0.11, min_child_weight=3.0, subsample=0.8, missing=nan, max_delta_step=0, max_depth=7  n_estimators=100, reg_alpha=1.0, reg_lambda=1, scale_pos_weight=3 |
| **SE-2**  **(Local Training)** | *Catboost* | learning_rate: 0.16, n_estimators: 125  l2_leaf_reg: 10.0, border_count: 50, max_depth: 12 |
| **SE-3**  **(Local Training)** | *XGBoost* | base_score=0.5, colsample_bylevel=1, colsample_bynode=1, colsample_bytree=0.45, gamma=0.9, importance_type='gain'  learning_rate=0.30, min_child_weight=3.0, subsample=0.85, missing=nan, max_delta_step=0, max_depth=7  n_estimators=75, reg_alpha=0.5, reg_lambda=1, scale_pos_weight=3 |
| **SE-4**  **(Local Training)** | *XGBoost* | base_score=0.5, colsample_bylevel=1, colsample_bynode=1, colsample_bytree=0.85, gamma=0.5, importance_type='gain'  learning_rate=0.11, min_child_weight=3.0, subsample=0.8, missing=nan, max_delta_step=0, max_depth=7  n_estimators=100, reg_alpha=1.0, reg_lambda=1, scale_pos_weight=3 |
| **SE-5**  **(Local Training)** | *Light GBM* | colsample_bytree=0.85, n_estimators=1200,  num_leaves=101, reg_alpha=0.25,  reg_lambda=0.087, scale_pos_weight=5 |
| **SE-6**  **(Local Training)** | *CatBoost* | learning_rate: 0.46, n_estimators: 50  l2_leaf_reg: 2.0, border_count: 50, max_depth: 8 |
| **NE-1**  **(Local Training)** | *XGBoost* | base_score=0.5, colsample_bylevel=1, colsample_bynode=1, colsample_bytree=0.45, gamma=0.9, importance_type='gain'  learning_rate=0.30, min_child_weight=3.0, subsample=0.85, missing=nan, max_delta_step=0, max_depth=7  n_estimators=75, reg_alpha=0.5, reg_lambda=1, scale_pos_weight=3 |
| **NE-2**  **(Local Training)** | *CatBoost* | learning_rate: 0.19, n_estimators: 150  l2_leaf_reg: 5.0, border_count: 200, max_depth: 10 |
| **NE-3**  **(Local Training)** | *Light GBM* | colsample_bytree=0.88, n_estimators=1067,  num_leaves=101, reg_alpha=0.42,  reg_lambda=0.87, scale_pos_weight=11 |
| **NE-4**  **(Other regions except specifc)** | *Light GBM* | colsample_bytree=0.85, n_estimators=1200,  num_leaves=101, reg_alpha=0.25,  reg_lambda=0.09, scale_pos_weight=5 |
| **MW-1**  **(All except specific)** | *XGBoost* | base_score=0.5, colsample_bylevel=1, colsample_bynode=1, colsample_bytree=0.85, gamma=0.7, importance_type='gain'  learning_rate=0.26, min_child_weight=2.0, subsample=0.80, missing=nan, max_delta_step=0, max_depth=7  n_estimators=225, reg_alpha=1.0, reg_lambda=1, scale_pos_weight=1 |
| **MW-2**  **(Same region except specific)** | *Light GBM* | colsample_bytree=0.88, n_estimators=1067,  num_leaves=101, reg_alpha=0.42,  reg_lambda=0.87, scale_pos_weight=11 |
| **MW-3**  **(Same region except specific)** | *XGBoost* | base_score=0.5, colsample_bylevel=1, colsample_bynode=1, colsample_bytree=0.85, gamma=0.5, importance_type='gain'  learning_rate=0.11, min_child_weight=3.0, subsample=0.80, missing=nan, max_delta_step=0, max_depth=7  n_estimators=100, reg_alpha=1.0, reg_lambda=1, scale_pos_weight=3 |
| **S-1**  **(Local Training)** | *CatBoost* | learning_rate: 0.39, n_estimators: 200  l2_leaf_reg: 4.0, border_count: 10, max_depth: 14 |
| **S-2**  **(Local Training)** | *Light GBM* | colsample_bytree=0.86, n_estimators=803,  num_leaves=147, reg_alpha=0.56,  reg_lambda=0.60, scale_pos_weight=1 |
| **S-3**  **(Same region except specific)** | *XGBoost* | base_score=0.5, colsample_bylevel=1, colsample_bynode=1, colsample_bytree=0.45, gamma=0.9, importance_type='gain'  learning_rate=0.30, min_child_weight=3.0, subsample=0.85, missing=nan, max_delta_step=0, max_depth=7  n_estimators=75, reg_alpha=0.5, reg_lambda=1, scale_pos_weight=3 |
| **N-1**  **(Reference plus same region absolute number)** | *Light GBM* | colsample_bytree=0.85, n_estimators=1200,  num_leaves=101, reg_alpha=0.25,  reg_lambda=0.09, scale_pos_weight=5 |
| **N-2**  **(All hospitals plus reference)** | *XGBoost* | base_score=0.5, colsample_bylevel=1, colsample_bynode=1, colsample_bytree=0.85, gamma=0.7, importance_type='gain'  learning_rate=0.26, min_child_weight=2.0, subsample=0.80, missing=nan, max_delta_step=0, max_depth=7  n_estimators=225, reg_alpha=1.0, reg_lambda=1, scale_pos_weight=1 |

**Supplementary Table 4 –** Calibration for each algorithm.

| **Hospital (Strategy)** | **Best algorithm** | **Scaled Brier Score** |
| --- | --- | --- |
| **SE-1 (Local Training)** | *XGBoost* | -0.04 |
| **SE-2 (Local Training)** | *Catboost* | 0.23 |
| **SE-3 (Local Training)** | *XGBoost* | 0.16 |
| **SE-4 (Local Training)** | *XGBoost* | 0.04 |
| **SE-5 (Local Training)** | *Light GBM* | 0.40 |
| **SE-6 (Local Training)** | *CatBoost* | -0.31 |
| **NE-1 (Local Training)** | *XGBoost* | -0.03 |
| **NE-2 (Local Training)** | *CatBoost* | 0.27 |
| **NE-3 (Local Training)** | *Light GBM* | 0.32 |
| **NE-4 (Other regions except specifc)** | *Light GBM* | -0.54 |
| **MW-1 (All except specific)** | *XGBoost* | -0.62 |
| **MW-2 (Same region except specific)** | *Light GBM* | -1.05 |
| **MW-3 (Same region except specific)** | *XGBoost* | 0.29 |
| **S-1 (Local Training)** | *CatBoost* | 0.24 |
| **S-2 (Local Training)** | *Light GBM* | 0.40 |
| **S-3(Same region except specific)** | *XGBoost* | -0.12 |
| **N-1 (Reference plus same region absolute number)** | *Light GBM* | -0.07 |
| **N-2 (All hospitals plus reference)** | *XGBoost* | 0.46 |


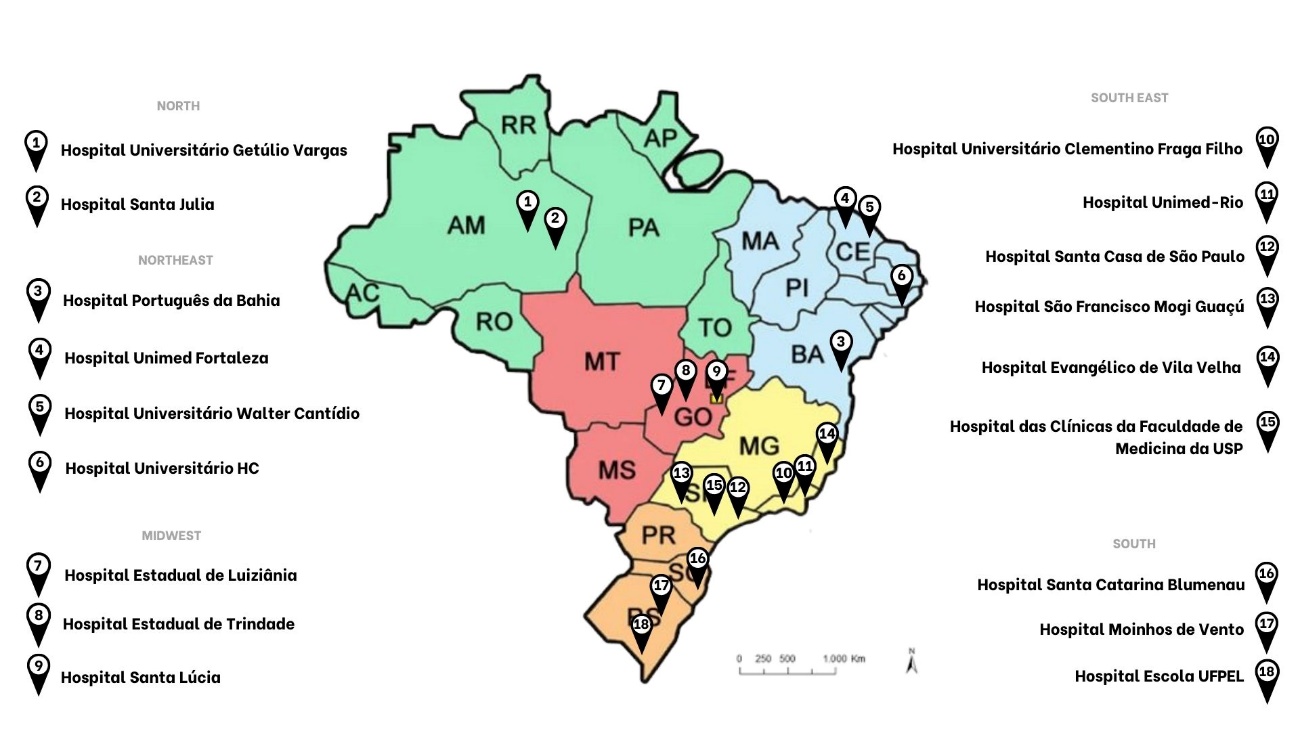


**Supplementary Figure 1.** Map of Brazil with the hospitals that participated in the study, according to geographic region.

Note: Figure was generated by the authors of this manuscript in Canva which is a free online graphic design tool. Please follow the link to access the original figure: <https://www.canva.com/design/DAFNoZ6To38/R6jZK32feOsblUaM9wKIFw/edit?utm_content=DAFNoZ6To38&utm_campaign=designshare&utm_medium=link2&utm_source=sharebutton>


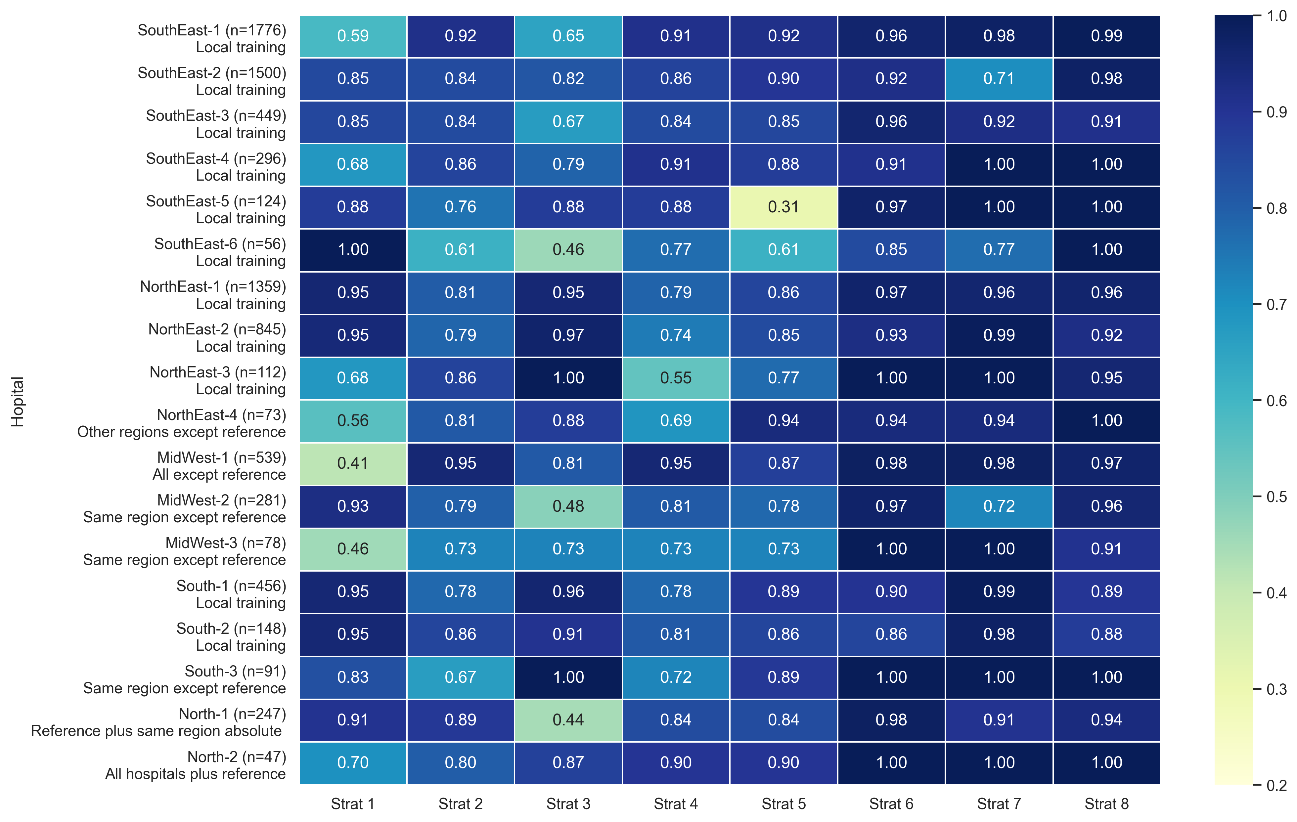


**Supplementary Figure 2.** Best specificities according to strategy, region and hospital with the best strategy highlighted.


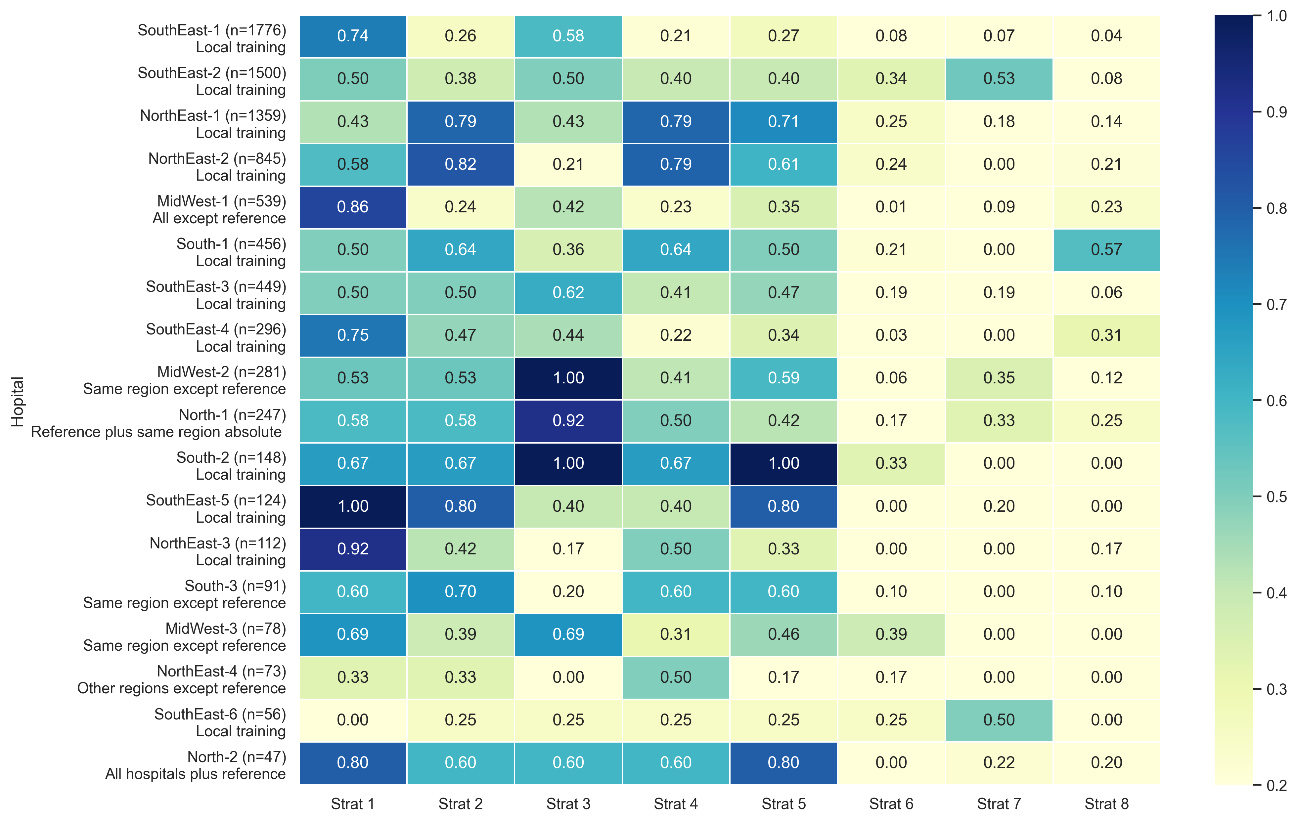


**Supplementary Figure 3.** Best recalls according to strategy, region and hospital with the best strategy highlighted.


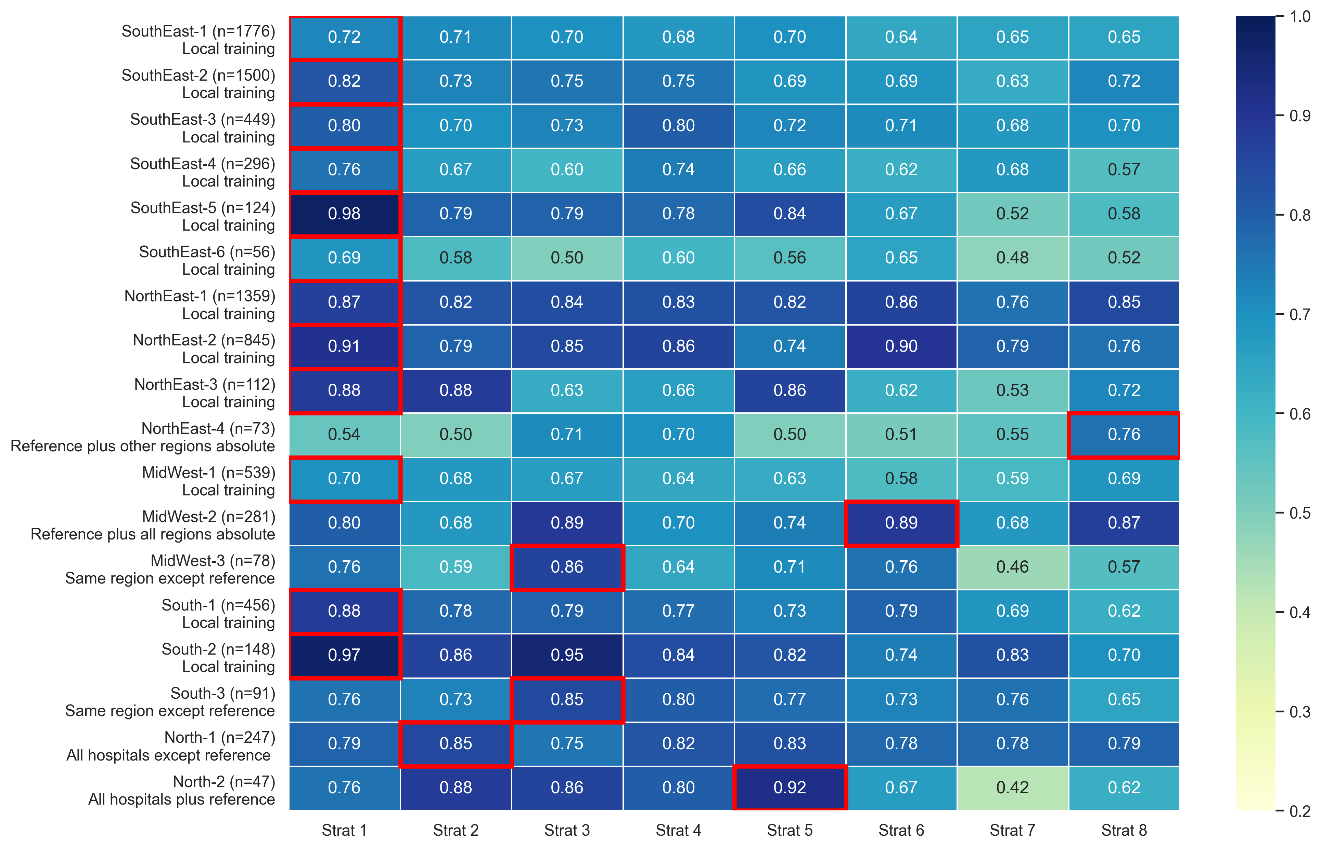


**Supplementary Figure 4.** Best AUROCs using the MICE imputation technique, with the region and hospital with the best strategy highlighted.
